# CD147 Aggravated Inflammatory Bowel Disease by Triggering NF-*κ*B-Mediated Pyroptosis

**DOI:** 10.1155/2020/5341247

**Published:** 2020-06-30

**Authors:** Zhaohui Xu, Ruitao Liu, Ling Huang, Yuxin Xu, Mingmin Su, Jiayu Chen, Lanlan Geng, Wanfu Xu, Sitang Gong

**Affiliations:** ^1^The First Affiliated Hospital of Jinan University, Jinan University, Guangzhou, China; ^2^Department of Gastroenterology, Guangzhou Women and Children's Medical Center, Guangzhou Medical University, Guangzhou, China; ^3^Department of Preventive Medicine, School of Public Health, Fujian Medical University, Fuzhou 350122, China; ^4^Department of Cancer Biology and Therapeutics, School of Pharmacy and Pharmaceutical Sciences, Cardiff University, Wales CF103AT, UK; ^5^Department of Neonatal Intensive Care Unit, Guangzhou Women and Children's Medical Center, Guangzhou Medical University, Guangzhou, China; ^6^Guangzhou Institute of Pediatrics, Guangzhou Women and Children's Medical Center, Guangzhou Medical University, Guangzhou, China

## Abstract

**Background:**

Pyroptosis, a novel form of inflammatory programmed cell death, was recently found to be a cause of mucosal barrier defect. In our pervious study, CD147 expression was documented to increase in intestinal tissue of inflammatory bowel disease (IBD).

**Objective:**

The aim of this study was to determine the function of serum CD147 in pyroptosis.

**Methods:**

The study group consisted of 96 cases. The centration of CD147, IL-1*β*, and IL-18 levels in serum was assessed by ELISA. Real-time PCR and WB were performed to analyze the effect of CD147 on pyroptosis.

**Results:**

In this study, our results showed that CD147 induced cell pyroptosis in intestinal epithelial cells (IECs) by enhancement of IL-1*β* and IL-18 expression and secretion in IECs, which is attributed to activation of inflammasomes, including caspase-1 and GSDMD as well as GSDME, leading to aggregate inflammatory reaction. Mechanically, CD147 promoted phosphorylation of NF-*κ*B p65 in IECs, while inhibition of NF-*κ*B activity by the NF-*κ*B inhibitor BAY11-7082 reversed the effect of CD147 on IL-1*β* and IL-18 secretion. Most importantly, serum CD147 level is slightly clinically correlated with IL-1*β*, but not IL-18 level.

**Conclusion:**

These findings revealed a critical role of CD147 in the patients with IBD, suggesting that blockade of CD147 may be a novel therapeutic strategy for the patients with IBD.

## 1. Introduction

It is well known that inflammatory bowel disease (IBD), including ulcerative colitis (UC) and Crohn's disease (CD) [1], is a chronic abnormal inflammatory disease of the gastrointestinal tract and remains the principal cause of cancer-related death in the world [2–4]. Imbalance between a proinflammatory reactive state and an anti-inflammatory reactive state is a critical cause in the development of intestinal barrier function, and the loss of immune system homeostasis is a hallmark of IBD [5–10].

Pyroptosis, a caspase-1-dependent programmed cell death, which features gasdermin D (GSDMD) cleavage and translocation and rapid plasma membrane rupture, results in the release of cytokines that activate proinflammatory immune cell mediators, including IL-1*β* and IL-18 [11–14]. Interestingly, IL-1*β*, one of the products of pyroptosis, has been reported to be an important mediator of inflammation and tissue damage in IBD, and IL-1*β* induced defects in intestinal epithelial tight junctions resulting in increased intestinal permeability [15], while IL-18 has been also shown to contribute to the breakdown of the mucosal barrier, triggering inflammation and amplifying damage elicited to the intestinal epithelium during disease [16]. What is more, the clinical studies have shown that correlation between increased epithelial secretion of IL-18 and increased severity of IBD suggests that IL-18 may play a key pathogenic role in inflammatory disorders, such as CD [17]. Mechanistically, there are two distinct signaling pathway-derived pyroptosis types, either canonical pyroptosis or noncanonical pyroptosis [18–20]. These findings showed that pyroptosis plays an importantly role in IBD. However, the critical molecule-mediated pyroptosis remains to be elucidated in IBD.

CD147, also named basigin, is a highly glycosylated transmembrane protein and is a potent inducer of matrix metalloproteinases (MMPs) and angiogenic factors such as vascular endothelial growth factor (VEGF) [21–23]. Recently, a study showed that CD147 plays an important role in driving brain inflammation after ischemic stroke by regulating oligodendrogenesis and white matter integrity [24], and inhibition of CD147 alleviated acute ischemic stroke by reducing thrombin inflammation [25]. What is more, the splenic inflammatory response induced by cerebral ischemia was inhibited by blocking CD147, suppressing the expression of cytokines (TNF*α*, IL-6, and IL-1*β*) and monocyte chemoattractant protein-1 (MCP-1) in the spleen after the ischemia onset [26]. These findings implied that CD147 has a potential role in inflammatory disease; however, there are no available reports about the function of CD147 in pyroptosis in IBD, which remains to be elucidated.

## 2. Materials and Methods

### 2.1. Patients and Sample Collection

Based on the declaration of Helsinki as reflected in a prior approval by the institution's human research committee, this study was approved by the Medical Ethical Review Board, named Scientific Research Committee of Guangzhou Women and Children's Medical Center. Written informed consent was given by the caregiver of the child for his clinical records used, which are not publicly available since the database is currently not anonymous and contains all patients' name; however, it could be available upon request.

We consecutively enrolled patients with endoscopically or histologically confirmed IBD. Patients were excluded if they had another diagnosed autoimmune disease. At recruitment, patients completed a self-administered survey, including questions about demographic information (age, gender), type of IBD (UC or Crohn's (CD)), self-reported disease activity at the time of encounter (remission, mild, moderate, or severe), duration of IBD (<1 year, 1-10 years, and >10 years), history of prior nutritional deficiencies (iron, vitamin B12, and vitamin D), and prior medication use (sulfasalazine, mesalamine, olsalazine, 6-mercaptopurine, azathioprine, methotrexate, cyclosporine, infliximab, adalimumab, certolizumab pegol, natalizumab, ciprofloxacin, metronidazole, prednisone, and budesonide). Disease characteristics were confirmed by a medical chart review. Subjects with discrepancies between survey results and chart review were excluded. Patients with inactive (*n* = 19) or active (*n* = 77) IBD were involved in this study. Serum samples were collected from these patients. The inactive IBD were used as the control group (control, *n* = 19).

### 2.2. Cell Culture and Treatment

HT-29, Caco-2, and HCT116 cells of IECs were cultured in DMEM supplemented with 10% FBS and maintained in a humidified incubator at 37°C and 5% CO_2_. For treatment, the recombinant human CD147 (BP4745) cytokine was purchased from Boster Company.

### 2.3. CCK-8 Assay

A Cell Counting Kit-8 (CCK-8) (Dojindo, Japan) assay was performed as described in our pervious study [27]. Briefly, cells were seeded into a 96-well plate at a concentration of 5 × 10^3^ cells/well. After treatment with CD147 at the indicated time, each well was incubated with 10 *μ*L CCK-8 in 90 *μ*L of culture medium. The cells were incubated for 1 h at 37°C, and absorbance was measured at 450 nm. The assays were performed in triplicate.

### 2.4. Lactate Dehydrogenase (LDH) Detection Assay

An LDH assay was performed as described in Yin et al.'s study [28]. The levels of LDH were determined by using the LDH Release Assay Kit (Beyotime, Shanghai, China) according to the manufacturer's protocol. Absorbance values were detected at 490 nm using a microplate reader (Thermo Fisher Scientific, Waltham, MA, USA). Each experiment was performed in triplicate.

### 2.5. Real-Time PCR

Total RNA was extracted using TRIzol (Invitrogen, Carlsbad, CA, USA) and converted to cDNA using the All-in-One™ First-Strand cDNA Synthesis Kit (GeneCopoeia™, FulenGen) and amplified by PCR using the All-in-One™ qPCR Mix (GeneCopoeia™, FulenGen) according to the manufacturer's instructions. Primer sequences were as follows: for IL-18, CATACGAATTCCATGGGCAAGCTTGAATCTAAATTA (sense) and CATATGGATCCGCTAGTCTTCGTTTTGAACAG (antisense); for IL-1*β*: 5′-AGCTACGAATCTCCGACCAC-3′ (sense) and 5′-CGTTATCCCATGTGTCGAAGAA-3′ (antisense); and for GAPDH: 5′-TGCACCACCAACTGCTTAGC-3′ (sense) and 5′-GGCATGGACTGTGGTCATGAG-3′ (antisense).

### 2.6. Western Blotting Analysis

Total protein was prepared in 2x SDS sample buffer and subjected to SDS-PAGE and transferred to a 0.22 *μ*m nitrocellulose transfer membrane. The membrane was blocked with 5% (*w*/*v*) milk in PBS/0.05% (*v*/*v*) Tween 20 and incubated with the indicated antibody overnight at 4°C followed by incubation with a horseradish peroxidase secondary antibody (Jackson ImmunoResearch) for 1 h at room temperature. Proteins were detected using enhanced chemiluminescence (PerkinElmer). The antibodies listed as follows: caspase-1 (A0964), NLRP3 (A5652), ASC (A1170), IL-18 (A1115), IL-1*β* (A19635), GSDMD (A17308), GSDME (A7432); alpha-tubulin (AC012), beta-actin (AC004), and p-p65 (Ser536) (AP0124), were from ABclonal Company.

### 2.7. Enzyme-Linked Immunosorbent Assay

Blood specimens were adequately centrifuged for extracting serum specimens. Resulting serum specimens were stored at -80°C until analysis. Serum levels of CD147, IL-18, and IL-1*β* were detected using a commercial kit according to the manufacturer's instructions, and CD147 (E-EL-H1606c), IL-18 (E-EL-H0253c), and IL-1*β* (E-EL-H0149c) were purchased from Elabscience Biotechnology Co. Ltd. (Texas, USA).

### 2.8. Statistical Analysis

All statistical analyses were performed using SPSS 22.0 (IBM Corp., Armonk, NY, USA). Data were expressed as the mean with standard deviation (SD). A one-sample *t*-test and ANOVA were used to analyze the difference of IL-18 and IL-1*β* mRNA levels, or ELISA was used. All statistical analyses utilized a 0.05 level of significance.

## 3. Results

### 3.1. CD147 Induced a Phenomenon of Pyroptosis

**Figure 1:**
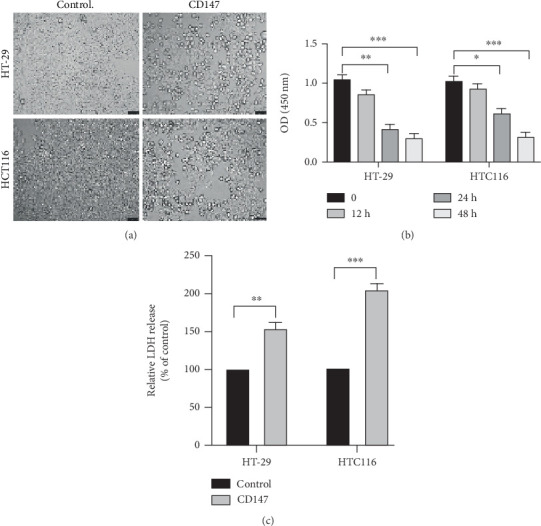
Effects of CD147 on pyroptosis. (a) Representative images of IECs (×200) treated with CD147 (10 ng/mL) for 24 hours. Scale bar = 100 *μ*m. (b) The CCK-8 assay was performed to detect cell viability in response to CD147 treatment for various time points. (c) The levels of LDH were measured by the LDH detection assay.

To explore the possible role of CD147 in pyroptosis, recombinant CD147 (10 ng/mL) was employed to treat IECs for 24 hours to monitor the phenomenon of pyroptosis. As shown in Figure 1(a), morphologically, a greater number of dead cells were observed in HT-29 and HCT116 cells after CD147 (10 ng/mL) stimulation compared with that untreated control group, respectively (Figure 1(a)). In addition, the results of CCK-8 analysis further revealed that cell viability was significantly reduced in response to CD147 treatment (Figure 1(b)). What is more, the levels of LDH were upregulated by CD147 stimulation (Figure 1(c)), implying that a greater number of dead cells were caused by CD147 treatment in HT-29 and HCT116 compared with the control group. Thus, these data indicated that CD147 could induce pyroptosis in IECs in vitro.

### 3.2. CD147 Triggered Pyroptosis to Aggravate an Inflammatory Reactive State

**Figure 2:**
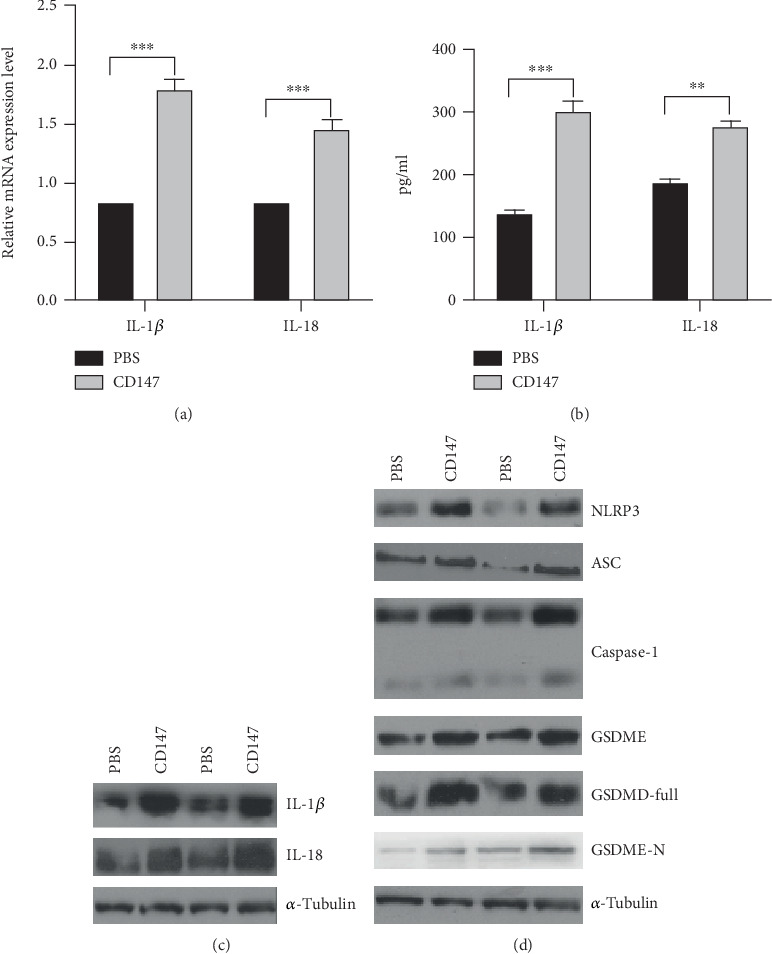
The changes of pyroptosis-related genes in response to CD147 treatment. (a) Real-time PCR and (b, c) western blotting were performed to analyze the changes of IL-18 and IL-1*β* expression. (d) Western blotting was used to detect NLRP3, ASC, caspase-1, GSDMD, and GSDME expression in the indicated group in HT-29 and Caco-2 cells. CD147 activated cell pyroptosis and contributed to IL-18 and IL-1*β* expression. CD147 induced pyroptosis-related gene expression in IECs.

Given that CD147 was shown to play a critical role for pyroptosis, we sought to identify the genes responsible for CD147-induced pyroptosis. Our results demonstrated that CD147 led to significant upregulation of IL-1*β* and IL-18 expression at the mRNA level by the real-time PCR assay and promote mature IL-1*β* and IL-18 expression by ELISA (Figures 2(a) and 2(b)). In line with this, the results from the western blotting have further confirmed that mature IL-1*β* and IL-18 expression was upregulated in IECs with CD147 treatment (Figure 2(c)).

As shown in Figure 2(d), our results demonstrated that CD147 treatment has led to a drastic enhancement of the core unit of the inflammasomes, such as NLRP3, caspase-1, and ASC at the protein level in HT-29 and Caco-2 cells, subsequently cleaving and activating caspase-1; consequently, activated caspase-1 cleaves and separates the N- and C-terminals of GSDMD [29]. Further results showed that CD147 treatment has markedly increased GSDMD and GSDME expression at the protein level (Figure 2(d)). Based on the above-mentioned findings, the critical function of CD147 in triggering pyroptosis, leading to IL-1*β* and IL-18 activation and maturation and aggravating intestinal inflammation.

### 3.3. Activation NF-*κ*B Pathway Involved in CD147-Induced Pyroptosis

**Figure 3:**
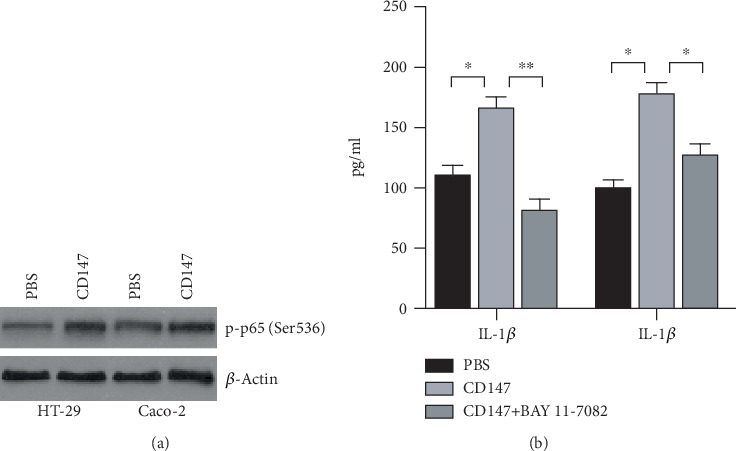
CD147 regulated IL-18 and IL-1*β* expression in an NF-*κ*B-dependent manner. (a) Western blotting was performed to examine the phosphorylation of NF-*κ*B in HT-29 and Caco-2 cells. (b) ELISA was employed to examine IL-18 and IL-1*β* secretion.

An increasing amount of research has revealed that the NF-*κ*B pathway has participated in NLRP3 inflammasome activation [30–32]. Attenuating inflammation protects patients against IBD, which leads us to seek the change of NF-*κ*B activity in response to CD147 treatment. As shown in Figure 3(a), phosphorylation of NF-*κ*B is increased in HT-29 and Caco-2 cells treated with CD147 when compared with the control group. Most importantly, inhibition of NF-*κ*B by the NF-*κ*B inhibitor BAY11-7082 significantly reversed the effect of CD147 on IL-1*β* and IL-18 expression by ELISA (Figure 3(b)). These findings suggested that CD147 contributed to IL-1*β* and IL-18 expression in an NF-*κ*B-dependent way.

### 3.4. Serum CD147 Is Clinically Correlated with IL-1*β* and IL-18

**Figure 4:**
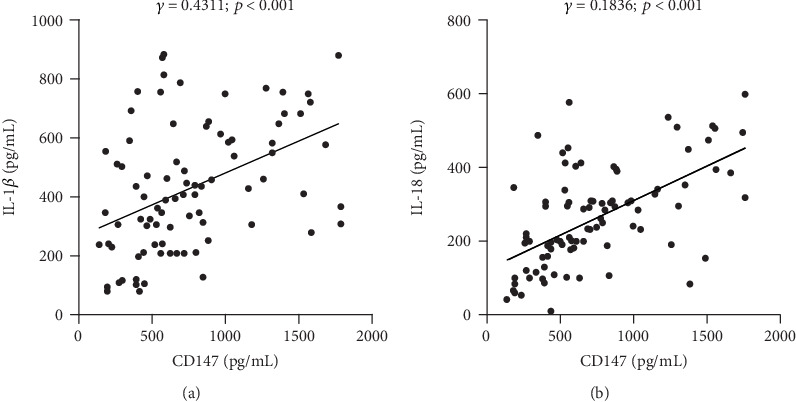
Clinical correlation between CD147 and IL-18 as well as IL-1*β*. ELISA was used to examine the level of CD147, IL-18, and IL-1*β* in serum from patients with inactive (*n* = 19) or active (*n* = 77) IBD. The clinical association was generated between CD147 and IL-1*β* (a) and IL-18 (b).

Since the above results showed that pyroptosis could be regulated by CD147, hence we next explored the possible clinical relationship between CD147 and IL-1*β* as well as IL-18, an end product of pyroptosis. Therefore, ELISA and statistical analysis were performed to analyze the level of IL-1*β*, IL-18, and CD147 in serum from the subjects with active or inactive IBD. As expected, the result showed a positively clinical association between CD147 and IL-1*β* (Figure 4(a), *R* = 0.4311, *p* < 0.001); in addition, the level of CD147 is slightly clinically correlated with IL-18 expression level (Figure 4(b), *R* = 0.1836, *p* < 0.001). These findings indicated that CD147 could be a novel mediator of an inflammatory regulator in IBD.

## 4. Discussion

An extracellular matrix metalloproteinase inducer (EMMPRIN), a highly glycosylated transmembrane protein of the immunoglobulin superfamily, was widely expressed on various cell types, including the brain, liver, spleen, intestine, and kidney, and especially in tumor cells [33–36]. It has been reported that the CD147 acts as a novel modulator in inflammation and immune responses [36]. Therapeutic targeting of CD147 has been shown to reduce inflammation and disease severity in experimental models of human diseases such as rheumatoid arthritis, asthmatic lung inflammation, myocardial ischemia/reperfusion injury, multiple sclerosis, and experimental autoimmune encephalomyelitis [37–41]. In our previous study, we have demonstrated that CD147 is increased in the intestinal mucosa of patients with IBD, which is correlated with DAI (unpublished data). Up to now, available reports about the function of CD147 in IBD are limited; herein, we showed that CD147 aggravated intestinal inflammation by the activation of pyroptosis and treatment of IECs with CD147 significantly promoted IL-1*β* and IL-18 expression and secretion in IECs, which is attributed to the activation of caspase-1, leading to the cleavage of pro-IL-1*β* and IL-18 into mature IL-1*β* and IL-18 and their release into the extracellular microenvironment via GSDMD and GSDME. Mechanically, CD147 treatment in IECs significantly increased the phosphorylation of p65, and inhibition of p65 overcame the effect of CD147 on mature IL-1*β* and IL-18 expression. These findings implied a novel insight into CD147-aggravated IBD by the activation of NF-*κ*B-mediated pyroptosis.

Pyroptotic cell death, also known as pyroptosis or inflammatory cell necrosis, was mediated by the gasdermin family and accompanied by inflammatory response [42–44]. Nucleotide-binding domain leucine-rich repeat family protein 3 (NLRP3) inflammasome activation was the key procedure of cell pyroptosis [45, 46]. Interestingly, maturation and secretion of IL-1*β*, IL-18, and IL-37 are mediated and required by inflammatory caspases within inflammasome signaling complexes [47, 48]. By inducing pyroptosis, specific IL-1 family cytokines are expressed by cells as cytosolic proforms that require cleavage for their activity and cellular release. IL-1*β*, IL-18, and IL-37 maturation and secretion are governed by inflammatory caspases within signaling platforms called inflammasomes [49]. In this study, we found that treatment of IECs with CD147 results in increased NLRP3/ASC/caspase-1/GSDMD expression, the core component of the inflammasome, and leads to maturation and secretion of IL-1*β* and IL-18, suggesting that CD147 is a novel mediator of inflammation in aggravating inflammatory reaction by the regulation of pyroptosis.

It is well known that one major signaling pathway is involved in the activation of the NLRP3 inflammasome. An NF-*κ*B-activating stimulus is required for cells to express pro-IL-1*β* and optimal NLRP3 [30]. In our study, after CD147 treatment, p-NF-*κ*B p65 was increased significantly while the total of NF-*κ*B p65 expression was unchanged; in addition, inhibition of NF-*κ*B activity by BAY11-7082 could drastically reverse the promotion of CD147 on IL-1*β* and IL-18, implying that CD147 regulated pyroptosis in an NF-*κ*B-dependent way. Most importantly, serum level of CD147 is clinically correlated with IL-1*β* and IL-18, respectively. However, further work is required to elucidate how CD147 regulated NF-*κ*B activity. In summary, these findings extended the function of CD147 and revealed a critical role of CD147 in the patients with IBD, suggesting that blockade of CD147 may be a novel therapeutic strategy for the patients with IBD.

## Data Availability

The data used to support the findings of this study are available from the corresponding author upon reasonable request, which is attributed to the unpublic clinical materials.

## Abbreviations

ASC: Apoptosis-associated speck-like protein containing a CARD
cDNA: Complementary DNA
CD: Crohn's disease
VEGF: Vascular endothelial growth factor
GSDMD: Gasdermin D
IBD: Inflammatory bowel disease
IECs: Intestinal epithelial cells
IL: Interleukins
MMPs: Metalloproteinases
MCP-1: Monocyte chemoattractant protein-1
NF-*κ*B: Nuclear factor kappa-light-chain-enhancer of activated B cells
NLRP3: NOD-like receptor family, pyrin domain-containing 3
qPCR: Quantitative polymerase chain reaction
TNF: Tumor necrosis factor
UC: Ulcerative colitis.
